# The *Cladophora glomerata* Enriched by Biosorption Process in Cr(III) Improves Viability, and Reduces Oxidative Stress and Apoptosis in Equine Metabolic Syndrome Derived Adipose Mesenchymal Stromal Stem Cells (ASCs) and Their Extracellular Vesicles (MV’s)

**DOI:** 10.3390/md15120385

**Published:** 2017-12-08

**Authors:** Krzysztof Marycz, Izabela Michalak, Ievgeniia Kocherova, Monika Marędziak, Christine Weiss

**Affiliations:** 1 Department of Experimental Biology, Wroclaw University of Environmental and Life Sciences, Chelmonskiego 38 C, 50-630 Wroclaw, Poland; kocherova.evgenia@gmail.com (I.K.); monika.maredziak@upwr.edu.pl (M.M.); 2 Wroclawskie Ctr Badan EIT, Stabłowicka 147 St, 54-066 Wroclaw, Poland; 3 Department of Advanced Material Technologies, Faculty of Chemistry, Wrocław University of Science and Technology, Smoluchowskiego 25 St, 50-372 Wrocław, Poland; izabela.michalak@pwr.edu.pl; 4 PferdePraxis Dr. Med. Vet. Daniel Weiss, Postmatte 14, CH-8807 Freienbach, Switzerland; d.weiss@horsedoc.ch

**Keywords:** algae extract, *Cladophora glomerata*, biosorption, trivalent chromium, EMS, ASCs

## Abstract

This study investigated in vitro effects of freshwater alga *Cladophora glomerata* water extract enriched during a biosorption process in Cr(III) trivalent chromium and chromium picolinate on adipose-derived mesenchymal stromal stem cells (ASCs) and extracellular microvesicles (MVs) in equine metabolic syndrome-affected horses. Chemical characterisation of natural *Cladophora glomerata* was performed with special emphasis on: vitamin C, vitamin E, total phenols, fatty acids, free and protein-bound amino acids as well as measured Cr in algal biomass. To examine the influence of *Cladophora glomerata* water extracts, in vitro viability, oxidative stress factor accumulation, apoptosis, inflammatory response, biogenesis of mitochondria, autophagy in ASCs of EMS and secretory activity manifested by MV release were investigated. For this purpose, various methods of molecular biology and microscopic observations (i.e., immunofluorescence staining, SEM, TEM, FIB observations, mRNA and microRNA expression by RT-qPCR) were applied. The extract of *Cladophora glomerata* enriched with Cr(III) ions reduced apoptosis and inflammation in ASCs of EMS horses through improvement of mitochondrial dynamics, decreasing of PDK4 expression and reduction of endoplastic reticulum stress. Moreover, it was found, that *Cladophora glomerata* and Cr(III) induce antioxidative protection coming from enhanced SOD activity Therefore, *Cladophora glomerata* enriched with Cr(III) ions might become an interesting future therapeutic agent in the pharmacological treatment of EMS horses.

## 1. Introduction

Equine metabolic syndrome (EMS) is an endocrine disease that is characterized by a multiplate pathophysiological condition including hyperinsulinemia and/or resistance to insulin (IR), past and/or chronic laminitis, hyperlipidemia as well as local and systemic inflammation [1,2,3]. Although, obesity and/or regional adiposity have been widely regarded as a characteristic feature and diagnostic parameter of EMS horses, recent findings suggest that obesity may become a marker indicating metabolic dysfunction, since not all obese horses suffer for EMS. Nevertheless, severe obesity and regional adiposity are strongly associated with increased morbidity and mortality in horses [3]. The major risk factors of EMS development, besides genetic predispositions, are unbalanced feeding protocol based on high starch and high carbohydrates diet in parallel with limited exercise, which lead to disturbances in insulin dynamics including fasting hyperinsulinemia and/or exaggerated insulin response to oral or intravenous glucose administration [4]. Nevertheless, excessive accumulation of adipose tissue surrounding the nuchal ligament (“cresty neck”) behind the shoulder or close to the tail head has been shown to generate pro-inflammatory cytokines and result in profound changes in cellular composition of fat tissue [1,5]. The abundantly infiltrated adipose tissue by macrophages, lymphocytes and mast cells, hypoxia of adipocytes, activation of inflammasomes, endoplasmic reticulum stress, free radicals and activation of TLR are finally responsible for meta-inflammation development [6,7,8] The adipokines released by adipocytes including leptin, resistin, adiponectin, visfatin and apelin cause many metabolic abnormalities and modulate immune responses [9]. The local inflammation of adipose tissue and abundant adipokines generation in fat tissue of EMS horses impairs other organ functions, including the cardiovascular system, that additionally complicated the patient picture [10]. The local inflammation, together with elevated oxidative stress of adipose tissue are not without molecular consequences for adipose-derived mesenchymal stem progenitor cells (ASCs) which reside within it [11]. These cells, because of their unique properties including mulitilineage differentiation potential, self-renewal and clonogenic potential (CFU-fs) and the ability to control tissue homeostasis are considered as promising therapeutic agents in many different equine veterinary fields including endocrine disease treatment [8,12]. However, the prolonged inflammation and insulin resistance of EMS horses, in particular over expression of pro-inflammatory cytokines, might drastically reduce ASC stemness and their anabolic activity, which is recognized as a regenerative potential [13,14]. ASCs possesses the ability to synthesise and secrete of broad range of growth factors that are transferred to neighbor cells through membrane-derived vesicles (MVs) [15]. Their molecular importance is additionally underlined by their ability to deliver functional mRNA and/or miRNA involved in ASC proliferative potential, viability and immunosuppressive effect—all crucial from EMS conditions’ point of view [16]. However, the ASC functionality and their regenerative properties are strongly limited in EMS horses by excessive adipose tissue inflammation combined with enhanced oxidative stress [17]. Recently, it has been shown that ASCs from EMS horses, because of elevated apoptosis, mitochondrial dysfunction and higher senescence, are important for their clinical application [16,17]. Moreover, it has been found that ASCs from EMS horses suffer from serious impairment of their osteogenic and chondrogenic differentiation potential. Although ASCs are exposed to unfavorable adipose tissue micro-environments, they developed macroautophagy and selective mitophagy to survive in that conditions.

The management of EMS mainly boils down to caloric restriction (restricted grass access, grazing muzzles, dry lots, etc.), feeding diets with a reduced percentage of non-structural carbohydrates and obesity control through exercise programs. The application of bioactive feed additives may become a promising tool in prevention and management of EMS horses. One of the most extensively investigated additives in insulin resistance (IR) as well as obesity management is trivalent chromium, or chromium picolinate, which is thought to improve glucose metabolism mechanisms of chromium in alleviating insulin resistance [18]. However, there is some controversial data indicating no effect on weight or insulin sensitivity in horses when fed with chromium for 16 weeks [19]. Thus, combining different sources of chromium including trivalent chromium and chromium picolinate with bioactive plant components in the course of biosorption processes might be a future solution for that problem. There is an increasing number of evidence that bioactive compounds found in seaweeds including *Cladophora glomerata* exert positive effects on health due to their antioxidative effects [20,21]. Algal biomass contains many groups of active compounds including vitamins, essential amino acids, minerals and fatty acids. Moreover, algae are characterized by high antioxidant activity that might effectively reduce free radicals and thus induce SOD activity in cells culture [22]. Besides beneficial chemical composition, *Cladophora glomerata* might be enriched with various minerals by the biosorption process, which increases its nutritional value and therapeutic properties. Biosorption is a process of passive sorption and complexation of particular sorbents including ions such as Cr(III), that besides their sorbent properties also become valuable nutrients [23,24]. Thus, the combination of biosorption, with particular of Cr(III) ions together with high content of antioxidants as well as anti-inflammatory compounds, might be a beneficial future diet for EMS treatment. In the present work, the effect of water extract obtained from freshwater alga *Cladophora glomerata* and enriched by biosorption process with Cr(III) ions and separate chromium picolinate on equine metabolic-affected ASCs was investigated. It was found that the combination of *Cladophora glomerata* and Cr(III) improves ASC viability, proliferative activity and induces anti-apoptotic and anti-inflammatory properties via shedding MVs from ASCs. Moreover, it was shown that applied water extract of *Cladophora glomerata* and Cr(III) lead to reduction of oxidative stress and simultaneously induces enhanced SOD activity. Obtained data suggest that the combination of chromium with *Cladophora glomerata* might become, in future, a reasonable component of diet-based therapy in EMS horses.

## 2. Results

### 2.1. Algae Content Analysis

Measurements of vitamin C content, performed in three different samples (*n* = 3), indicated mean content of 21.7 mg/100 g of dry algae. The content of α-tocopherol was 2.5 mg/100 g of dry algae, whereas β- and γ-tocopherol were not detected. Concentration of polyphenols was on average 196 mg/100 g of the sample dry algal biomass. Results of fatty acid content in *Cladophora glomerata* biomass are presented in Table 1 as mg/100 g of dry algal mass. Composition of free and protein-bound amino acids in *C. glomerata* is presented in Table 2 (expressed as mg/100 g of dry mass).

### 2.2. Immunophenotyping and Multipotency of ASCs

**Figure 1:**
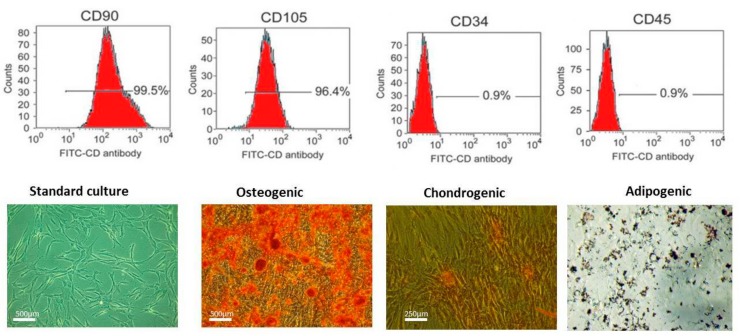
Phenotypical characterization of adipose-derived mesenchymal stem cells (ASCs). Isolated cells were positive for CD90 and CD105. ASCs are not characterized by having surface markers for CD45 and the hematopoietic lineage marker CD34. Multipotency assays indicated the ability of cells to differentiate into osteogenic (Alizarin red staining), chondrogenic (Safrain O staining) and adipogenic (Oil red O) lineages.

The analysis of ASCs indicated a heterogenous cells populations including stem cells. Figure 1 shows that the investigated population expressed the following markers: CD90, CD105 and were negative for CD45 and CD34 which excluded their hematopoietic origin (Figure 1). Multipotency assays indicated that isolated cells were able to differentiate into three lineages: osteogenic, adipogenic and chondrogenic.

### 2.3. Cell Viability

**Figure 2:**
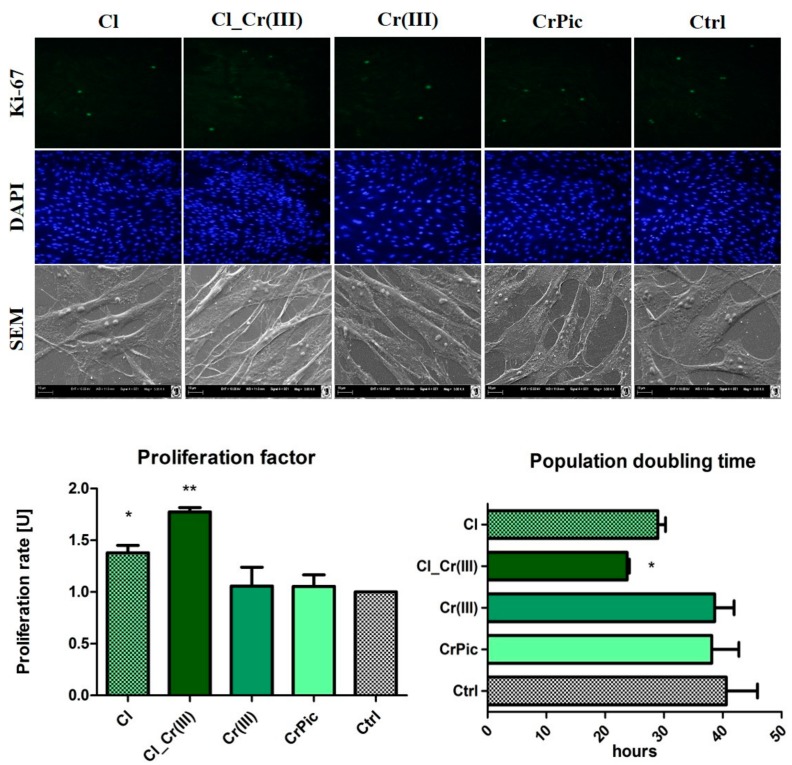

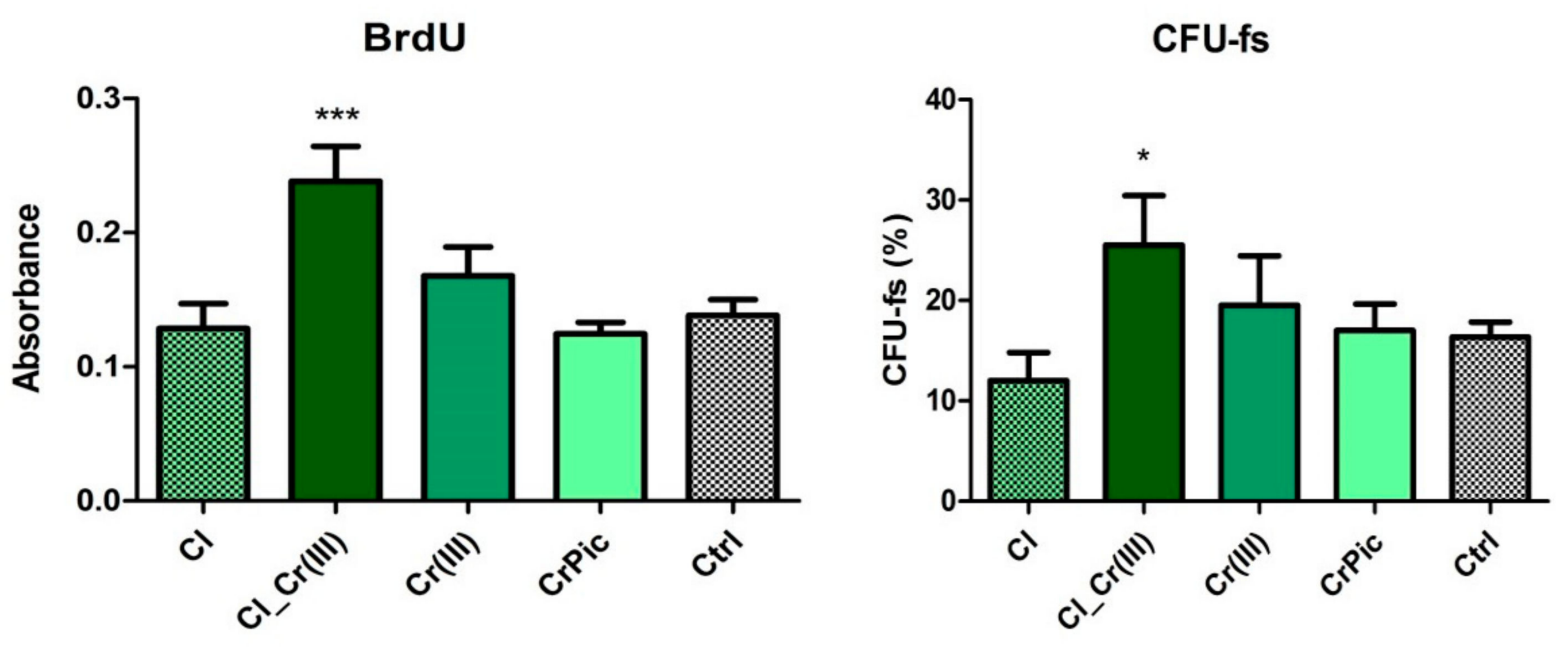
Proliferation activity of ASCs. Results of proliferation factor, population doubling time, BrdU and ability of cells to form colonies in groups treated with different extracts in comparison to control. Representative photographs showing the results of Ki67 and DAPI staining and SEM photographs. Results expressed as mean ± SD. * *p* < 0.05, ** *p* < 0.01, *** *p* < 0.001.

**Figure 3:**
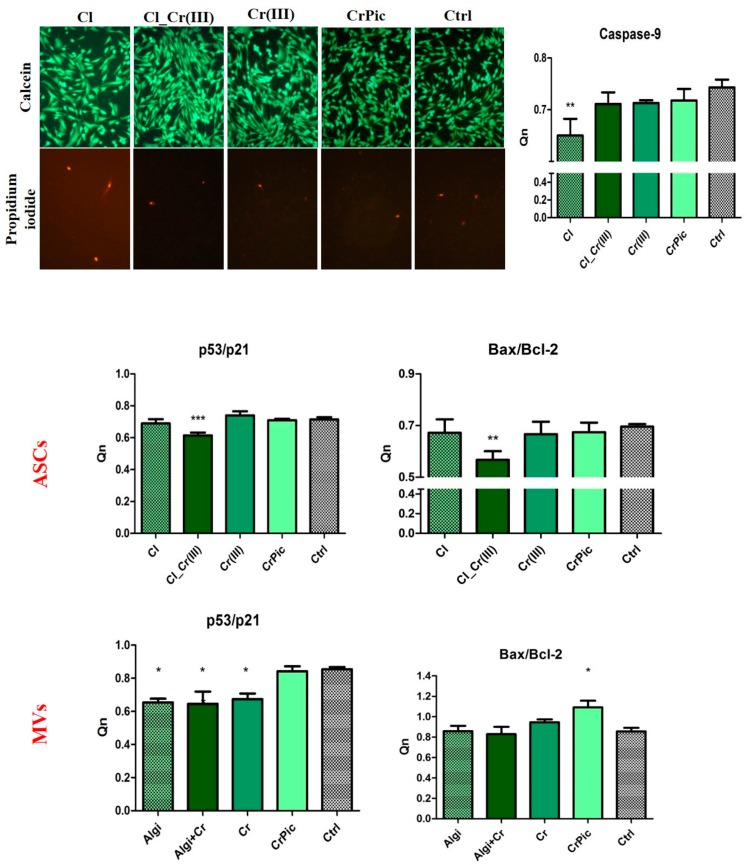
Representative photographs of live dead cells using Calcein and propidium iodide staining. Expression of genes involved in apoptotic pathways in ASCs and MVs treated with bioactive extracts. Results expressed as mean ± SD. * *p* < 0.05, ** *p* < 0.01, *** *p* < 0.001.

To compare cell viability with different doses of obtained extracts a 48-h preliminary viability test was performed. Cell number after 48-h incubation indicated that 0.5 μM concentration was considered as the most positive, thus, further experiments were performed using that concentration. Next, the proliferation assays based on the cell metabolic activity were performed. Compared to the control (Ctrl), greater proliferation activity of EqASCs, was observed in the cultures supplemented with extract of *Cladophora glomerata* (Cl) and *Cladophora glomerata* enriched with Cr(III) ions [Cl_Cr(III)]. The viability of EqASCs was not changed by supplementation with pure Cr(III) and CrPic (Figure 2). As predicted, time required to double the population-PDT was greatest in the Cl_Cr(III) group in comparison to the control. Furthermore, in this group, the highest ability of cells to form colonies originated from one cell. Analysis of miRNA expression revealed significant downregulation of mir-145 in the Cl_Cr(III) group. Additionally, BrdU analysis revealed increased incorporation into cells during their division in groups cultured with both *Cladophora glomerata* with Cr(III). As a consequence, a lower activity of Ki-67 staining in the *Cladophora glomerata* group and *C. glomerata* and Cr(III) group in comparison to the control was observed. The highest expression of Ki-67 protein was in in the Cr(III) group. Live dead assay based on Calcein AM and propidium iodide staining indicated an elevated number of dead cells in the control group when compared to *Cladophora* groups (Figure 3). p53/p21 and Bax/Bcl-2 ratio were significantly decreased in ASCs cultured with *Cladophora*_Cr(III) extracts when compared to the control. Interestingly, the same gene expression measured in MVs isolated from ASCs cultured with bioactive compound indicated a decreased ratio of p53/p21 in *Cladophora*, *Cladophora*_Cr(III), Cr(III) in comparison to the control. Bax/Bcl-2 expression increased in CrPic group of MVs (Figure 3).

### 2.4. Morphology of Cells and Mitochondrial Biogenesis

**Figure 4:**
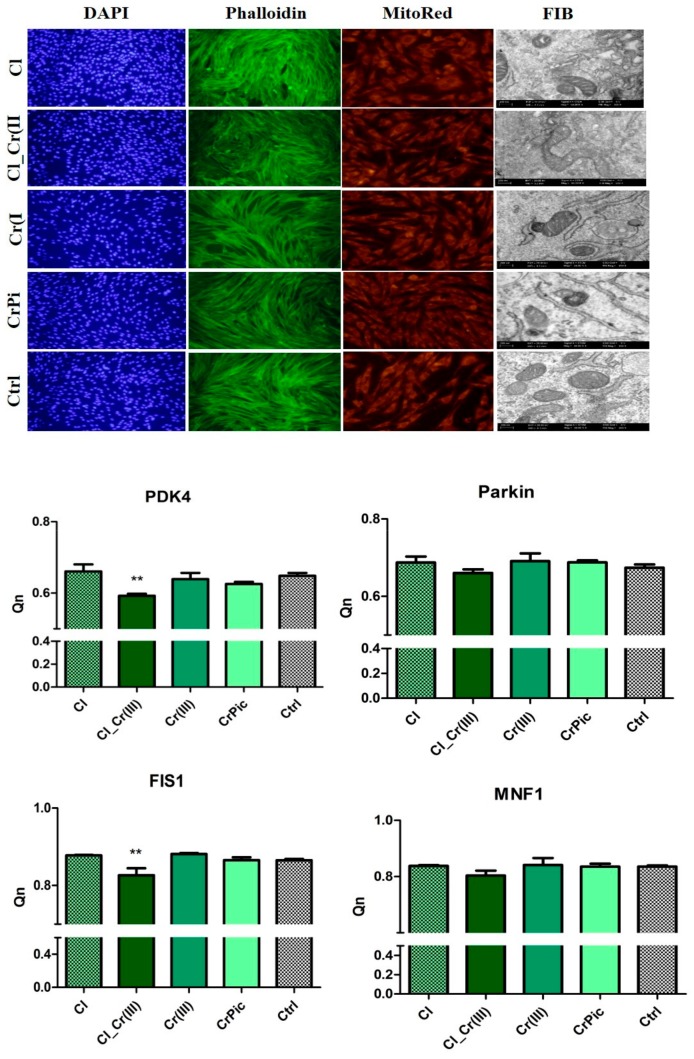
Representative photographs of DAPI, Phalloidin and MitoRed staining. FIB microphotographs showing mitochondria of cells. The expression of mitochondria biogenesis-related genes-PDK4, Parkin, FIS1, MNF1. Results expressed as mean ± SD. * *p* < 0.05, ** *p* < 0.01, *** *p* < 0.001.

Morphological differences between cells cultured with bioactive compounds investigated in this study are shown in Figure 4. The addition of different extracts did not negatively affect cell morphology, and cells exhibited proper spindle shape morphology with bipolar shape. However, in experimental cultures (except CrPic) intracellular connections were better developed than in control group. TEM observations indicated that mitochondria of cells cultured with Cr(III), CrPic and ASCsEMS cultured without additives were visible swollen in comparison to those cultured with *Cladophora glomerata* and *Cladophora glomerata* with Cr(III). Next, the expression of genes responsible for mitochondrial biogenesis were performed. The expression of parkin slightly decreased in the Cl_Cr(III) group in comparison to control and other groups. However, results are not statistically significant. To examine mitochondrial dynamics, the effects of FIS and MNF expression on mRNA level were investigated. Expression of FIS and PDK4 was significantly decreased in the *Cladophora glomerata* CL_Cr(III) group. On the contrary, no significant differences between the investigated groups in case of MNF expression was found.

### 2.5. Endoplasmic Reticulum (ER) Stress and Autophagy

**Figure 5:**
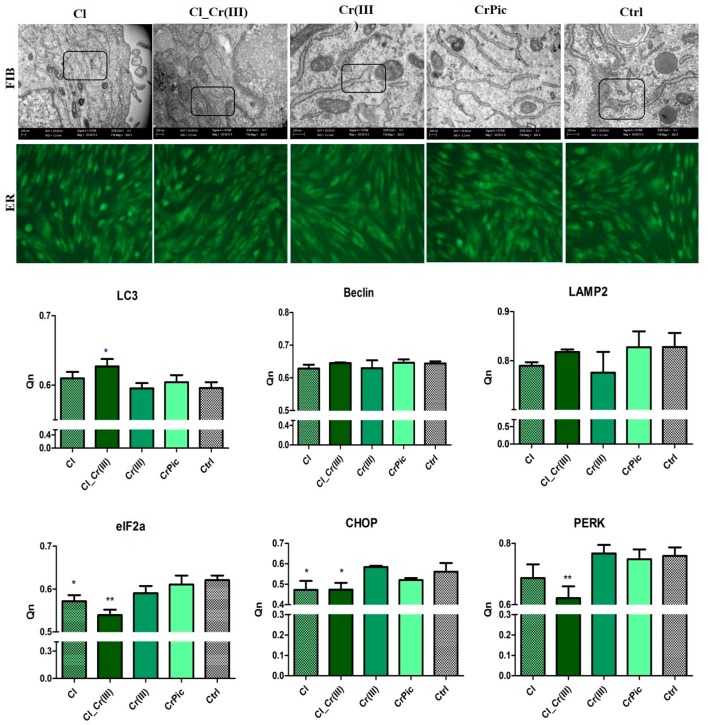
Expression of LC-3, Beclin, LAMP2, eIF2a, CHOP, PERK measured using qRT-PCR. Representative photographs showing ER by TEM observations and fluorescent staining. Results expressed as mean ± SD. * *p* < 0.05, ** *p* < 0.01, *** *p* < 0.001.

TEM, fluorescence microscopy and qRT-PCR were used to determine autophagy. Cells cultured with *Cladophora glomerata* and both *Cladophora glomerata* enriched with Cr(III), the ER was well-developed whereas in other groups, ER was enlarged, fragmented and disintegrated (Figure 5). Furthermore, large, double-membranous autophagic vacuoles and pre-autophagosome structures were observed. qRT-PCR results confirmed ER stress in control cells as well as ASCs cultured with Cr(III), which was confirmed by elevated expression of CHOP and PERK. Interestingly, mRNA levels of LC3 increased only in the Cl_Cr(III) group. Expression of eIF2a was decreased in *Cladophora* and Cl_Cr(III) groups, whreas addition of bioactive compounds did not influence expression of beclin and LAMP2 transcripts.

### 2.6. Secretory Activity

**Figure 6:**
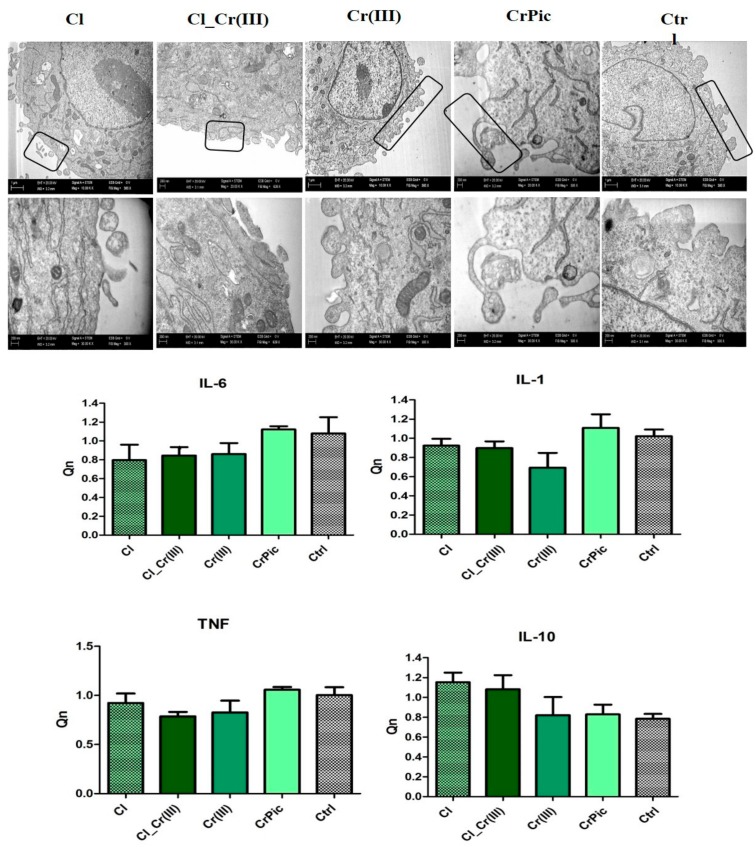
Pro- and anti-inflammatory genes expressed in MVs. Representative TEM microphotographs presenting secretion of MVs. Results expressed as mean ± SD. * *p* < 0.05, ** *p* < 0.01, *** *p* < 0.001.

TEM observations reveled, that cells cultured with investigated extracts abundantly secreted membrane-derived microvesicles (MVs). The release of MVs was more apparent in Cl group, whereas the number of MVs secreted by the control group was reduced (Figure 6). Interestingly, no significant differences in expression of Il-1, IL-6 and TNF-alpha mRNA levels in all experimental groups were found, however the levels of pro-inflammatory proteins (IL-1, IL-6, TNF-alpha) were slightly increased in CrPic and control groups.

## 3. Discussion

Equine metabolic syndrome (EMS) becomes a more and more frequent endocrine disorder in horses, and is caused by high caloric feeding strategies combined with limited exercise and genetic predisposition [4,25]. Not treated or not prevented, EMS usually leads to laminitis which is often followed by equine euthanasia. Thus, searching for an effective treatment on nutrition, cellular and/or combined levels (cellular therapies) seem to be fully reasonable [16,26]. Algae extracts have been widely reported to possess many beneficial properties, including therapeutic effects in arthritis, cardiovascular diseases and diabetes treatment [27,28,29,30]. Here, we carried out a comparative analysis for the evaluation of *Cladophora glomerata* water extract alone and that enriched by a biosorption process in Cr(III) as well as trivalent chromium and chromium picolinate on ASCs derived from EMS horses. In this study, we used ASCs isolated from EMS horses specific for stem cell surface markers. The cells used in this research exhibited multilinege differentiation potential and thus fulfilled criteria of multipotency and plasticity as well as mesenchymal stem progenitor cell definition.

**Figure 7:**
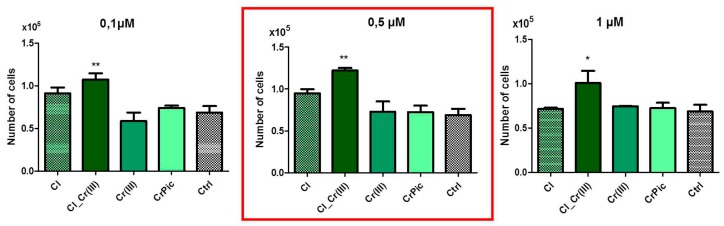
Screening test based on proliferative activity of cells cultured with compounds investigated in this experiment. Results expressed as mean ± SD. * *p* < 0.05, ** *p* < 0.01, *** *p* < 0.001.

In this study, 0.5 µM *Cladophora glomerata* water extract was used on the basis of preliminary screening tests, which showed that this concentration was most beneficial when ASC proliferative activity was considered (Figure 7). Among all tested groups, within five-days proliferative assays, ASCs treated with Cl_Cr(III) extract exhibited the highest proliferation rate with simultaneous shortened population doubling time (PDT), and were characterized by the highest percentage of Ki-67 positive cells. Moreover, in these group, the highest ability of ASCs to form single colonies (CFU-fs) was observed. Additionally, the expression of mir-145, which is known to suppress stem cell self-renewal, was significantly downregulated in the Cl_Cr(III) group. Obtained data clearly indicate that the combination of Cl together with Cr(III) significantly improves ASC viability and proliferative activity. The observed effect might be due to high concentrations of bioactive compounds e.g., amino acids, fatty acids or polysaccharides, that have been previously found and well-documented in Cl. The abundance of polysaccharides in *Cladophora glomerata* might explain their pro-proliferative effect on ASCs influencing mitochondrial function and thus secretion proteins involved in apoptotic pathways [31]. Recently, it was shown that EMS-derived ASCs are affected by higher apoptosis, reduced viability as well as seriously impaired mutipotency when compared to healthy ASCs. We found, that ASCs from EMS horses treated with Cl_Cr(III) extract improves their viability through decreased expression of p53, p21 transcript with simultaneous improvement of BAX/BCL2. Interestingly, similar cytophysiological effects on MVs derived from ASCs treated with Cl_Cr(III) extract was observed. These shed a promising light on using extract, since MVs are recognized as vesicles, that transferred information on mRNA/miRNA level to neighbouring cells and thus might improve microenvironmenta of local tissues including adipose tissue. Bearing in mind the fact that adipose tissue in EMS horses becomes a source of local and systemic inflammation, the MVs transfer anti-apoptotic mRNA/miRNA and might regulate apoptosis in target cells which is highly desirable. Moreover, in ASCs, the Cl_Cr(III) extract induced realising of microvesicles (MVs) containing down regulated anti-inflammatory transcripts including IL-1, IL-6 as well as TNF-α. The down regulation on mRNA level (of most proinflammatory cytokines) indicates anti-inflammatory effects of tested extract. Obtained data might be explained by the presence of phenols and pheohytin in green algae, that have been shown to posses anti-inflammatory activity [32]. Furthermore, beside pro-inflammatory agents, the expression of anti-inflammatory cytokines within MVs was examined. It was found that the up-regulation of both IL-10 and TGF-β in Cl_Cr(III) has anti-inflammatory activity in isolated MVs. The simultaneous anti-inflammatory, as well as immunomodulatory, effect of Cl_Cr(III) might be explained by high concentrations of polyphenols as well as C-phycocyanin (C-PC) [33]. Moreover, organic chromium complexes were recently shown to affect chronic inflammation and diabetes type II [34]. Thus, the combination of *Cladophora glomerata* enriched in chromium(III) in biosorption processes might become a reasonable strategy to not only reduce systemic inflammation but also induce immunomodulatory effects, which are all crucial in EMS horses. Moreover, antioxidative and anti-senesces effects of Cl_Cr(III) when compared to the control cells were observed. The ASCs treated with Cl_Cr(III) exhibited reduced accumulation of β-galactosidase, reduced ROS and NO levels with simultaneous higher anti-oxidative defence coming from SOD activity. In contrast to the Cl_Cr(III) group, control cells were characterized by the enlarged cell bodies and nuclei. The observed effect seems to be fundamental in future potential applications of Cl_Cr(III) in EMS horses, since we previously showed that EMS is characterized by elevated ROS levels with significantly reduced SOD activity. The anti-oxidative effect of Cl_Cr(III) might result from high concentrations of chlorogenic acid, rutin or cryptochlorogenic acid that were reported to reduce ROS as well as NO levels. Interestingly, obtained data showed that poor Cr(III) reduced ROS and NO and induced SOD activity. Obtained data stands in good agreement with those of Patlolla and colleagues, who found increased activities of SOD and CAT in liver and kidney in hexavalent-induced chromium in Sprague–Dawley rats [35]. In this study, reduced PDK4 and FIS1 expression (mRNA level) in ASCs treated with Cl_Cr(III) was observed. It was found that inhibition of PDK4 increases glucose tolerance in high-fat-diet, insulin-resistant mice [36]. Moreover, the reduced expression of PDK4 shed a promising light on the therapeutic potential of Cl_Cr(III) since the elevated expression of PDK is characteristic of patients with diabetes, vascular calcification and heart failure [37]. In turn, it has been shown that diabetes mellitus and metabolic syndrome could alter mitochondrial dynamics by increased release of reactive oxygen species (ROS) and accelerated mitochondrial fission [38]. Here, we observed down regulation of FIS1 mRNA among other tested groups, indicating mitochondrial dynamic improvements [39]. Moreover, transmission electron microscopy investigations allowed mitochondria cristae and their improvement in ASCs treated with Cl_Cr(III) in contrast to abundant mitochondria found in other groups. In parallel to mitochondrial improvement, the reduction of endoplasmatic reticulum stress (ER) which is recognized in EMS-derived progenitor cells as an autophagy inducer affecting the recycling of organelles was found. As was previously demonstrated, diabetes-disrupted ER stress initiates pancreatic β-cell failure and apoptosis. Here, the regulation of PERK mRNA levels in ASCs treated with Cl_Cr(III) was observed. It might be due to anti oxidative activity of Cl_Cr(III), which could improve ER function. 

Recently, bioactive compounds that might find an application in equine metabolic syndrome are gaining significant interest. Here, the beneficial effect of Cl_Cr(III) on EMS-derived progenitor cell proliferative activity, viability and oxidative stress were observed. The used Cl_Cr(III) extract reduces apoptosis and inflammation in ASCs of EMS horses through improvement of mitochondrial dynamics, decreasing of PDK4 expression and reduction of ER stress. Thus, Cl_Cr(III) might become an interesting therapeutic agent in future clinical application.

## 4. Materials and Methods 

The study was conducted after the acceptance of the the II Local Ethics Committee of Environmental and Life Sciences University (Chelmonskiego 38C, 51-630 Wroclaw, Poland; decision No. 84/2012). Chemicals used in these experiment were purchased from Sigma Aldrich (Taufkirchen, Germany) unless otherwise specified.

### 4.1. Bioactive Compounds Preparation 

The biomass of freshwater macroalgae, *Cladophora glomerata,* was collected from the surface of the pond in Tomaszówek (51°27′21′′ N 20°07′43′′ E) in October 2016.

The biosorption of Cr(III) ions by *Cladophora glomerata* was performed according to the procedure described by Michalak and Chojnacka [40]. The powder of natural and *Cladophora glomerata* ions enriched with Cr(III) was minced using mine-thrower, dissolved in a sterile water–methanol solution (20% methanol) and incubated overnight. The obtained mixture was then filtrated 5 times with 0.45 µm pore size filter and incubated in a water-bath (40 °C) to evaporate methanol. The final concentration of chromium in the extract prepared from *Cladophora glomerata* enriched with Cr(III) ions by the biosorption process was 10 µM. Then, by adding appropriate amounts of extract directly to the culture medium, we obtained three different concentrations of chromium—0.1 µM, 0.5 µM and 1 µM.

### 4.2. Characteristics of Cladophora glomerata Biomass

#### 4.2.1. Determination of Vitamin C Content in *Cladophora glomerata*

Content of vitamin C in *Cladophora glomerata* was measured using the 2,6-dichlorophenolindophenol (DIP) method. To quantify measurements of ascorbic acid, a standard curve was prepared in a concentration range of 0–2 mg/100 mL. Aliquots of *C. glomerata*. (0.5 g) were extracted with 10 mL of extraction mixture (5 g of oxalic acid, 0.75 g of Na2EDTA in 1 L) using an ultrasonic extractor for 5 h at room temperature, and subsequently homogenized. The obtained mixture was filtered with 0.45 µm membrane filters. DIP dye (0.02 mg/mL) was added in 5:1 (*v*/*v*) ratio. The absorbance was measured spectrophotometrically at 520 nm.

#### 4.2.2. Gas Chromatographic Assay of Tocopherol Content

Vitamin E (α-, β-, γ-, δ-tocopherol; Tc) content was measured using GC/FID chromatography with squalene as internal standard. For the investigation, 0.5 g of dry alga was mixed with 10 mL of 10% NaCl, 20 mL of hexane, 10 mL of methanol, and 10 µL of squalene solution (1.2 mg/mL) for 24 h at room temperature, and then sonicated for 1 h. Hexane phase was separated by centrifugation and evaporated to dryness. The remains were dissolved in 25 µL of hexane, and 2 µL of the prepared sample was placed in an injection chamber. The conditions of the analysis were as follows: Injection chamber temperature 250 °C; split 1:50; passive gas: He, flow-5 mL/min; column Varian VF-5 ms, 30 m × 0.53 mm; temperature: isothermal 110 °C/0.2 min, linear gradient 30 °C/min to 140 °C (1 min), linear gradient 10 °C/min do 230 °C (9 min), isothermal 230 °C for 6 min, linear gradient 10 °C/min to 300 °C (10 min) isothermal 300 °C for 7 min; detector: FID, detector temperature: 340 °C. The limit of the detection was equal to 0.1 mg/100 g. 

#### 4.2.3. Determination of the Total Phenols Content (TPC)

Algal biomass (0.5 g) was shaken for 1 h in darkness with 10 mL of 80% aqueous solution of methanol adjusted to a pH 1.5. The suspension was subsequently transferred to a homogenizer and homogenized for 1 min. The precipitate was centrifuged (5 min, 6000 rpm) and the supernatant was subjected to the further analysis. Total phenols content was determined spectrophotometrically. In brief, sample was added into Folin–Ciocalteu reagent (1:1) and incubated for 3 min. Saturated aqueous solution of Na_2_CO_3_ was added, incubated for 90 min in the darkness to settle and then filtered (pore size 0.45 μm). The absorbance was measured at 780 nm using quartz cuvette. The phenolic content was expressed as mg of gallic acid equivalent (GAE) per 100 mg of the sample.

#### 4.2.4. Extraction and Quantification of Fatty Acids

Total lipids were extracted from *Cladophora glomerata* dry biomass with 10 mL of Folch mixture. Extraction was performed for 24 h and sonicated for 1 h. Then precipitate was filtered out using PTFE membrane (0.45 μm) to recover liquid phase. The solvent was evaporated under vacuum, and the filtrate was dissolved in 0.5 mL of MTBE. Internal standard solution (methyl undecanoate in the volume of 25 µL; 43 mg/10 mL MTBE) and TMSH (0.25 mL) in 0.2 M methanol was subsequently added. The analysis was performed using a Varian 450-gas chromatograph (GC) equipped with a flame ionization detector (FID). The fatty acids were separated in a Varian VF-WAXms column (30 m × 0.53 mm × 1 µm film thickness). Column temperature was programmed as follows: initial temperature was set at 50 °C (2 min), then raised from 50 °C to 250 °C at a rate of 10 °C/min, maintained at 250 °C for 23 min. Detector and injection chamber temperatures were set at 250 °C. Helium was used as a carrier gas at 1 mL/min flow. Limit of the detection was equal to 0.1% weight.

#### 4.2.5. Analysis of Free and Protein-Bound Amino Acids

Mixing 1 g of dry alga with 10 mL of 1 M hydrochloric acid for 24 h at room temperature was performed to extract free amino acids. Internal standard solution (norVal; 11 mg/10 mL) was added, the suspension was homogenized and precipitate separated by centrifugation. 0.1 mL of the extract was evaporated at 60 °C, 0.05 mL of acetonitrile and 0.05 mL of functionalizing reagent (MTBSTFA + 1% TBDMSCl) was added. The obtained mixture was heated at 100 °C for 1 h. Protein hydrolysis was carried out by heating 10 mg of investigated biomass with 1 mL of 6 M HCl at 110 °C. In order to enable analysis of acid-sensitive amino acids, hydrolysis in the presence of 1% phenol was additionally performed [41]. After completion of hydrolysis, 20 µL of the samples was collected, 10 µL of the standard (norVal, 11 mg/10 mL) was added, the mixture was evaporated (60 °C), and functionalization procedure was performed as described above. Quantitative analysis was performed by GC/FID using Varian VF-5ms column (30 m × 0.53 mm × 0.5 µm film thickness). Helium was used as a carrier gas at a constant flow of 5 mL/min. The oven temperature program was set as follows: 5 min at 170 °C, heating at 4 °C/min up to 200 °C, maintenance at 200 °C for 3 min, increase to 300 °C at 4 °C/min, held at 300 °C for 20 min. Injection chamber and detector temperatures were 250 °C and 300 °C, respectively.

#### 4.2.6. Determination of Cr(III) in *Cladophora glomerata* Biomass

Deionized Milli-Q water (Millipore, Burlington, MA, USA) was used for the solution preparation. As standard solution Cr(III) (20 g/L) was used. Eluent was prepared by using 33 g of ammonium sulphate dissolved in 500 mL of water. Then, 30% NH_4_OH was added, mixed and transferred in a volumetric flask. Samples were filtered through 0.45 μm filters and through Dionex on Guard-P syringe cartridges before injection. For analysis, A DX-500 Chromatography System (including GP40 Gradient Pump or IP20 Isocratic Pump, AD20 Absorbance Detector, PC10 Postcolumn Pneumatic Delivery Package, Thermo Scientific™ Dionex, OnGuard-P Sample Pretreatment Cartridges) was used.

### 4.3. Animals Qualifications

Equine adipose tissue was obtained from 6 horses (both sexes; age range of 9–14 years; 11.2 ± 1.7 years). Adipose tissue from tail head region under septic condition was collected according to previously described protocol [14]. In the course of horses’ qualification following criteria were used: (i) extensive interviews with owners; (ii) measurement of body weight; (iii) estimation of body condition score (BCS) and cresty neck scoring system (CNS); (iv) palpation and visual assessment of the hoof capsule; (v) X-ray examination; (vi) resting insulin levels; (vii) combined glucose-insulin test (CGIT); and (viii) LEP concentration as previously described by Basinska et al. [13].

### 4.4. Isolation and Immunophenotyping of Adipose-Derived Stromal Cells

Equine adipose tissue samples were obtained from the tail base area, under local anesthesia induced by 2% lidocaine (Polfa S.A., Warsaw, Poland). Obtained tissue biopsies were washed using Hanks’ Balanced Salt Solution (HBSS) with the addition of 1% antibiotic to exclude microbial contamination. After that the tissue was cut using surgical scissors and digested with collagenase type I solution (0.1 mg/mL) for 40 min in CO_2_ incubator (37 °C, 5% CO_2_). After centrifugation (1200× *g*, 10 min) supernatant was discarded, and the cell pellet was resuspended in medium and seeded in cell culture flask.

The phenotype of equine ASCs was determined by FACS analysis using fluorochrome conjugated monoclonal antibodies against: CD44, CD45, CD90 and CD105.

### 4.5. Propagation of Equine Adipose Derived Mesenchymal Stromal Cells

For primary equine stem cells Dulbecco’s Modified Eagle’s Medium (DMEM) with Nutrient F-12 was applied, whereas secondary cell culture was maintained with DMEM containing 4500 mg/L of glucose (both supplemented with 10% of FBS and 1% of antibiotic-antimycotic solution). Cells were kept in 37 °C, 5% of CO_2_ incubator. 

### 4.6. Cell Viability

EqASCs proliferative factor was determined using TOX8 assay. Cells were incubated with medium containing 10% of dye (in CO_2_ incubator for 2 h). After incubation spectrophotometric measurement of samples was performed at wavelength of 600/690 nm using microplate reader (Spectrostar Nano, BMG Labtech, Ortenberg, Germany). Viability of cells was expressed as arbitrary unit. Values above 1 were considered as increase metabolic activity while decrease as value under 1. Additionally, time required to double population (PDT) was determined using a population doubling time on-line calculator.

Cell proliferation was also assessed with the thymidine analog BrdU (5-bromo-2′-deoxyuridine). To determine dye incorporation into newly synthesized DNA, BrdU Cell Proliferation (colorimetric) (abcam, Cambridge, UK) was performed according to producent instruction. Cells were cultured at a density 5 × 10^3^ cells/100 μL/well. Absorbance measurement using a microplate reader (Spectrostar Nano, BMG Labtech) at dual wavelength 450/550 nm quantified the reaction product.

The number of viable and dead cells was determined using Cell Double Staining Kit (Sigma Aldrich, Taufkirchen, Germany). Dead cells were stained with propidium iodide, whereas the viable with Calcein-AM. Cells were then observed using epifluorescence microscopy. The number of dead cells was calculated as percentage of all viable cells.

### 4.7. Ability of Cells to Form Colonies (CFU-Fs)

To perform colony fibroblast unit assays (CFU-Fs), cells were cultured in six-well plates at the density 1 × 10^3^ cells/well. After 7 days, cells were fixed (4% paraformaldehyde) and stained with pararosaniline (Sigma Aldrich). CFU-Fs was calculated by dividing number of colonies (consisted of more than 50 cells) by initial cell number. Score was expressed in percent.

### 4.8. Immunofluorescence Staining

Prior to staining, cells were fixed and permeabilized in PFA and 0.5% Triton X-100 for 20 min at room temperature, washed three times with PBS and blocked with 10% Goat Serum, 0.2% Tween-20. Incubation with primary antibody against Ki67 (Abcam) diluted 1:200 in PBS containing 1% Goat Serum and 0.2% Tween-20 was performed. Before secondary antibody was added (mouse anti-rabbit secondary antibodies conjugated with atto-488, dilution 1:200), cells were washed with PBS avoiding direct light. Finally, cells nuclei were stained by DAPI (1 µg/mL) for 5 min.

### 4.9. Morphology Evaluation

EqASCs morphology was observed using fluorescence (Zeiss, Axio Observer A.1) and scanning electron microscope (SEM, Zeiss Evo LS 15). The mitochondria were stained using MitoRed, nuclei with DAPI and cytoskeleton using atto-488-labeled phalloidin. Cells were fixed with 4% PFA (except those for MitoRed staining) and permeabilised with 0.1% Triton X-100. Before SEM observations post-fixed cells were dehydrated in ethanol and sputtered with gold and observed using SE1 detector, at 10 kV of filament tension. Prior to TEM observations, cells were fixed (2.5% glutaraldehyde for 24 h at 4 °C), washed three times and stained with 1% osmium tetroxide and lead citrate/uranyl acetate. Samples were dehydrated in acetone, embedded using an Agar Low Viscosity Resin Kit (Agar Scientific Ltd., Essex, UK), sectioned into ultrathin slices (70 nm), followed by collecting on copper gridsand observed using FE-STEM Auriga60 at 20 kV filament tension.

### 4.10. Microvesicles Isolation

Microvesicles were isolated from 3 × 10^8^ cells grown at 70% confluency in T75 flasks cultured in medium supplemented with FBS without exsosomes. Before isolation, number of cells was assessed using a 0.1% trypan blue exclusion test. Medium was collected and centrifuged (10 min, 200 g) followed by a 0.45 µm and 0.2 µm filter filtration. The obtained mixture was centrifuged once 30 min at 100,000 g and 4 °C and next-1 h at 40,000 g (centrifuge Optima XPN-, 100 Ti rotor, Beckman Coulter). Obtained MVs pellet was resuspended in 100 µL of PBS.

### 4.11. Analysis of mRNA and microRNA Expression—RT qPCR

Total RNA was isolated using Chomczyński method [42]. Using PrimeScript kit (Takara, Clontech) gDNA digestion and cDNA synthesis were performed for 150 ng of total RNA. The qRT-PCR was performed using SensiFast SYBR and Fluorescein Kit (Bioline) and 500 nM primers. CFX Connect™ Real-Time PCR Detection System (BioRad) was used. Results were calculated as arbitrary unists in reference to the housekeeping gene expression (GAPDH). To determine miRNA expression, after genomic DNA digestion with DNase I RNase-free kit (Thermo Fisher Scientific, Warsaw, Poland), 375 ng of total RNA was reverse-transcribed using Mir-X miRNA First-Strand Synthesis Kit (Clontech Laboratories, Inc., Mountain View, CA, USA). Next, the obtained cDNA was used for quantitative RT-PCR (final volume 25 μL) with SYBR Advantage qPCR Premix (Clontech Laboratories, Inc.). The level of miRNA expression was calculated in relation to U6 snRNA. The sequences of primers used are listed in the Table 3. 

### 4.12. Statistical Analysis

Statistical analysis was performed using GraphPad Prism 5.0 (San Diego, CA, USA). Statistical significance was determined using one-way analysis of variance (ANOVA) with Tukey’s post-hoc multiple comparison test. A *p* <0.05 was considered as statistically significant.

## Figures and Tables

**Table 1:** Fatty acids content of *Cladophora glomerata*. Results are expressed as the average of triplicate determinations ± standard deviation, where each of the following numbers were rounded to two significant figures. 8:0: caprylic acid, 10:0: capric acid, 12:0: lauric acid, 14:0: myristic acid, 14:1 (*n*-5): myristoleic acid, 15:0: pentadecylic acid, 16:0: palmitic acid, 16:1 (*n*-7): palmitoleic acid, 18:0: stearic acid, 18:1 (*n*-12): coenzyme A, 18:2 (*n*-6): linoleic acid, 18:3 (*n*-3): alpha-linolenic acid, 18:3 (*n*-6): γ-linolenic acid, 18:4 (*n*-3): stearidonic acid, 20:0: arachidic acid, 20:2 (*n*-6): eicosadienoic acid, 22:0: behenic acid.

Fatty Acid	Content [mg/100 g of Dry Mass]
8:0	0.50 ± 0
10:0	0.30 ± 0.10
12:0	0.20 ± 0.060
14:0	83.8 ± 0.50
14:1 (*n*-5)	0.50 ± 0.050
15:0	1.0 ± 0.20
16:0	160 ± 1.0
16:1 (*n*-7)	30 ± 0.20
18:0	4.8 ± 0.55
18:1 (*n*-12)	34 ± 0.26
18:2 (*n*-6)	14.23 ± 0.35
18:3 (*n*-3)	23 ± 1.1
18:3 (*n*-6)	n.d.
18:4 (*n*-3)	30 ± 0.55
20:0	0.80 ± 0.10
20:2 (*n*-6)	n.d.
22:0	4.0 ± 0.68

n.d.—not detected.

**Table 2:** Composition of free and protein-bound amino acids in *Cladophora glomerata* (mg/100 g of dry mas).

Amino Acid	Free	Bound
Ala	27.3 ± 2.5	1523.3 ± 70.2
Glc	9.3 ± 2.0	1313.3 ± 73.7
Val	0	1163.3 ± 55.0
Leu	4.3 ± 2.0	1503.3 ± 35.1
Ile	5.0 ± 1.7	826.6 ± 35.1
Asn	0	1496.6 ± 159.5
Asp	6.0 ± 1.7	1496.6 ± 159.5
Gln	0	1820.0 ± 70.0
Glu	0	1820.0 ± 70.0
Lys	0	560.0 ± 45.8
Arg	0	923.3 ± 15.3
His	0	90.0 ± 10.0
Phe	2.6 ± 1.2	916.6 ± 65.0
Tyr	0	743.3 ± 32.1
Trp	0	91.6 ± 16.0
Ser	14 ± 0.0	730.0 ± 43.6
Thr	0	596.6 ± 32.1
Met	0	230.0 ± 10.0
Cys	0	91.6 ± 7.6
Pro	6.6 ± 0.6	813.3 ± 32.1

**Table 3:** Sequences of primers used in qPCR.

Gene	Primer	Sequence 5′-3′	Amplicon Length	Accession No.
GAPDH	F:	GATGCCCCAATGTTTGTGA	250	NM_001163856.1
	R:	AAGCAGGGATGATGTTCTGG		
p53	F:	TACTCCCCTGCCCTCAACAA	252	U37120.1
	R:	AGGAATCAGGGCCTTGAGGA		
p21	F:	GAAGAGAAACCCCCAGCTCC	241	XM_003365840.2
	R:	TGACTGCATCAAACCCCACA		
Bcl-2	F:	TTCTTTGAGTTCGGTGGGGT	164	XM_014843802.1
	R:	GGGCCGTACAGTTCCACAA		
BAX	F:	GCCAGCAAATTGGTGCTCAA	94	XM_005596728.1
	R:	AGCAGTCACTTCCATGGCTC		
Casp9	F:	CACCTTCCCAGGCTTTGTCT	224	XM_014836232.1
	R:	GGCTCTGGCCTCAGTAAGTT		
PDK4	F:	GCTGGTTTTGGTTATGGCTTGC	137	XM_014853326.1
	R:	TCCACAGACTCAGAAGACAAAGCC		
Parkin	F:	TCCCAGTGGAGGTCGATTCT	218	XM_005608126.2
	R:	CCCTCCAGGTGTGTTCGTTT		
FIS	F:	GGTGCGAAGCAAGTACAACG	118	XM_014854003.1
	R:	GTTGCCCACAGCCAGATAGA		
MNF	F:	AAGTGGCATTTTTCGGCAGG	217	XM_001495170.5
	R:	TCCATATGAAGGGCATGGGC		
LC3	F:	TTACTGCTTTGCTCTGCCAC	213	XM_005608485.2
	R:	AGCTGCTTCTCCCCCTTGT		
Beclin	F:	GATGCGTTATGCCCAGATGC	147	XM_014729146.1
	R:	ATCCAGCGAACACTCTTGGG		
Lamp2	F:	GCACCCCTGGGAAGTTCTTA	139	XM_014733098.1
	R:	TTCGAGGATCTGTGCCAATCA		
CHOP	F:	AGCCAAAATCAGAGCCGGAA	272	XM_014844003.1
	R:	GGGGTCAAGAGTGGTGAAGG		
PERK	F:	GTGACTGCAATGGACCAGGA	283	XM_014852775.1
	R:	TCACGTGCTCACGAGGATATT		
eIF2 alpha	F:	AGCCAGAAGCCACTGCTAAG	196	XM_001488848.5
	R:	TGCACCTCCACCAGTTTGTT		
IL-6	F:	CGTCACTCCAGTTGCCTTCT	225	XM_014830631.1
	R:	GCCAGTACCTCCTTGCTGTT		
IL-1	F:	TATGTGTGTGATGCAGCTGTG	352	XM_014852743.1
	R:	ACTCAAATTCCACGTTGCCC		
IL-10	F:	GCGCTGGAGTTCCATCTCTT	196	XM_014739408.1
	R:	GCCTAGGTCCAAAGGGACAA		
TNF-alpha	F:	AAGTGACAAGCCTGTAGCCC	254	XM_014831605.1
	R:	GGTTGACCTTGGACGGGTAG		
